# Plant Development and Organogenesis: From Basic Principles to Applied Research

**DOI:** 10.3390/plants8090299

**Published:** 2019-08-24

**Authors:** Giovanna Frugis

**Affiliations:** Istituto di Biologia e Biotecnologia Agraria (IBBA), Unit of Rome, Consiglio Nazionale delle Ricerche (CNR), Via Salaria Km. 29,300, Monterotondo Scalo, 00015 Roma, Italy; giovanna.frugis@cnr.it

**Keywords:** plant development and organogenesis, translational research, crop productivity, genetic improvement, *Arabidopsis thaliana*, regulatory networks, phytohormones, *rol* genes, plant cell and tissue culture

## Abstract

The way plants grow and develop organs significantly impacts the overall performance and yield of crop plants. The basic knowledge now available in plant development has the potential to help breeders in generating plants with defined architectural features to improve productivity. Plant translational research effort has steadily increased over the last decade, due to the huge increase in the availability of crop genomic resources and *Arabidopsis*-based sequence annotation systems. However, a consistent gap between fundamental and applied science has yet to be filled. One critical point is often the unreadiness of developmental biologists on one side, to foresee agricultural applications for their discoveries, and of the breeders on the other, to exploit gene function studies to apply candidate gene approaches when advantageous. In this Special Issue, developmental biologists and breeders make a special effort to reconcile research on basic principles of plant development and organogenesis with its applications to crop production and genetic improvement. Fundamental and applied science contributions interwine and chase each other, giving the reader different but complementary perpectives from only apparently distant corners of the same world.

I am very pleased to introduce this Special Issue, which aims at reconciling research on basic principles of plant development and organogenesis with its applications to crop production and genetic improvement. This issue is published in honor of Domenico Mariotti, who significantly contributed to building up the Italian research community in Agricultural Genetics and Biotechnology and carried out the first experiments of *Agrobacterium*-mediated plant genetic transformation and regeneration in Italy during the 1980s. Domenico never believed in a clear distinction between fundamental and applied science; this is shown by his many scientific contributions to the field of cellular and molecular biotechnology in plants of agricultural interest spanning from basic to applied research. The review from De Paolis et al. [1] is dedicated to him, and summarizes the recent advances obtained in plant biotechnology and fundamental research following Mariotti scientific interests as guiding principles. Most of these themes recur throughout the Special Issue, where specific papers deepen into basic principles of developmental transitions and organogenesis, giving them a perspective in applied research and crop genetic improvement.

When we called for this Special Issue we were not prepared to such a prompt and enthusiastic response from the many friends/colleagues working in basic or applied research. We received many excellent manuscripts that made a major effort in forecasting translational solutions to improve crop production while addressing and reviewing fundamental knowledge of key plant developmental processes in model species [2,3,4,5,6]. Important contributions also came from researchers working on crop species [7,8] and plant breeding companies [6,9] that decided to openly share their strategies with the scientific community.

## 1. Key Questions in Root Developmental Biology and Target Genes for Root Crop Design

Di Mambro et al. [10] addressed the central question in developmental biology on how the body plan is established and maintained in multicellular organisms using *Arabidopsis* root as a simple model to study the molecular mechanisms of proximodistal and radial axes formation. The review describes all the main pathways and genes involved in establishing the two axes of growth in *Arabidopsis*, highlighting the involvement of some common players in controlling both axes and calling for more research in crop species in which root development shows higher levels of complexity [10]. Radial axis patterning is established by a finely regulated mechanism that controls the biosynthesis and activity of the phytohormone cytokinin, which in turn regulates auxin distribution and signaling. In another recent article, Di Mambro et al. have shown that cytokinin/auxin (CK/AUX) crosstalk is also involved in the regulation of root meristem size [11]. Cytokinins shape an auxin gradient by promoting the expression of *GH3.17*, which encodes an auxin-conjugating enzyme, in the most external layer of the root to position an auxin minimum in the last meristematic cells of the root to trigger cell differentiation [11]. In this Special Issue, Pierdonati et al. [12] from the same research group demonstrated that two additional *GH3* genes are expressed in the root, and also contribute to cytokinin-dependent positioning of the auxin minimum for root meristem size regulation. Fraudentali et al. showed how the CK/AUX-driven basic developmental frame can be taken over by reactive oxygen species (ROS) and other hormones signaling under stress conditions in the *Arabidopsis* plant model [13]. Leaf wounding triggers leaf to root long-distance communication resulting in early root xylem differentiation independent from root growth or meristem size. Root architecture and phenotypic plasticity influence crop productivity by affecting water and nutrient uptake, especially under environmental stress. These studies pave the way to unravel how long-distance communication may mediate phenotypic plasticity to adapt to changing environmental and stress conditions through the modification of the basic pathways of development [13].

The basic principles of root vascular development, provascular tissue formation and xylem differentiation, are described in the article from Hellmann et al. [4] where the key genetic pathways of primary and secondary development of *Arabidopsis thaliana* root are extensively reviewed, together with vascular development in shoot and hypocotyls. In this work the authors also focus on how this knowledge can and has been applied to agronomically important plants for production of wood and edible tubers as storage organs, providing important strategies and ideas to improve cambial activity in these processes [4].

The many regulatory candidate genes and pathways that are currently available in the *Arabidopsis* model are ready to be tested in crop biology and represent a valuable tool to be explored in breeding programs for root architectural traits.

## 2. Highjacking Plant Developmental Plans: The Case of the *Agrobacterium Rhizogenes Rol* Genes

In the review from De Paolis et al. [1] two sections are dedicated to the “hairy root” syndrome induced by *Agrobacterium rhizogenes*, characterized by the emergence of adventitious roots at the wound site of infected plants, and application of *A. rhizogenes* rooting locus (*rol*) genes to fruit tree propagation and transformation. How these *rol* genes act to highjack somatic plant cells to induce root meristem initiation and maintain indeterminate adventitious root growth is still a fascinating “enigma” after more than 30 years since their identification. However, evidence exists that they may act through the modification of as-of-yet unknown enzymatic reactions in the metabolism/signaling of cytokinins, auxin, and gibberellins as well as in ROS signaling [1]. In light of the current deep knowledge on root meristem formation and maintenance in *Arabidopsis*, it would be interesting to study the effect of *rol* genes in this model system to eventually identify their candidate target genes and pathways and understand their mode of action.

Trovato et al. [14] present a brief historical survey on the *rol* genes focusing on *rolD*, the only well characterized *rol* gene encoding an ornithine cyclodeaminase, which converts ornithine into proline. This type of enzyme is not present in plants, which synthesize proline through a more complex two-step reaction. The review illustrates how converging studies on *rolD* and proline function allowed to assess proline involvement in different plant developmental processes such as root elongation, flowering time, embryo formation, and pollen fertility. These studies corroborate the idea that different *rol* genes may act by interfering with plant metabolic pathways by encoding enzymes that bypass or redirect basic biochemical pathways. Since proline also acts as redox buffer and ROS scavenger, different *rol* genes may share a common role in the homeostasis of reactive oxygen species that can act as signaling molecules to regulate cellular processes underlying development [14].

## 3. Know the Old SAM: The Shoot Apical Meristem as the Key Developmental Switch in the Roadmap to Crop Yield Optimization

Three fascinating reviews guide the readers into the shoot apical meristem (SAM) world, where cells have to decide whether to keep on staying indeterminate (stem cells) or start the cell differentiation journey leading to the formation of complex organs such as leaves, flowers, and fruits. Several developmental features of plants, such as overall plant architecture, leaf shape, and vasculature architecture, that are major agricultural traits, depend on the activity of the SAM. The optimization of such developmental traits thus has great potential to increase biomass and crop yield. The failure of organizing a proper SAM in the embryo was also suggested to be involved in the post-zygotic incompatibility of wheat–rye hybrids [8].

The review of Fletcher [2] clearly summarizes the molecular mechanisms involved in stem cell maintenance in shoot and floral meristems through the molecular negative feedback loop called the CLAVATA (CLV)–WUSCHEL (WUS) pathway (CLV–WUS), both in the *Arabidopsis* model plant and crop species such as tomato, rice, and maize, highlighting similarities and specificities. Fletcher also illustrates the several examples of increased yield traits due to CLV–WUS pathway modulation in crop domestication, and foresees the great opportunity of using genome editing to enhance yield traits in a wide variety of agricultural plant species by fine-tuning the highly conserved CLV–WUS system [2].

The review of Traas [15] focuses on the basic principles guiding lateral organ formation at the shoot apical meristem, particularly on how auxin-dependent pathways can modulate wall structure to set particular growth rates and growth directions. How the molecular activity is translated into changes in geometry for oriented growth of organs and tissues is still unknown. The author brings the readers at the intersection of transcriptional regulation, mechanical forces and complex feedbacks from the cytoskeleton and the cell wall on gene expression, critically discussing the many questions that remain open in the field [15].

Richardson and Hake [3] consider another fascinating aspect of organogenesis at the shoot apical meristem, the formation of boundaries between pluripotent meristematic cells and differentiating organs. Their review critically summarizes the current understanding of boundary specification during vegetative development in grass crops in comparison with eudicot models. Gene regulatory networks (GRNs) underlying meristem/organ boundaries, as well as genetic modules that have been co-opted to specify within-organ boundaries to generate morphological diversity, are deeply analyzed in both eudicots and grass crops [3]. These GRNs are driven by different classes of transcription factors, the most important of which are NAC domain (NAM/ATAF/CUC), LBD (lateral organ boundaries domain), and KNOTTED1-like homeobox (KNOX) transcription factors (TFs). A specific section in De Paolis et al. [1] is also dedicated to KNOX TFs. Since boundary specification have a profound effect on leaf shape and plant productivity, GRN-based strategies to exploit this knowledge for crop genetic improvement are suggested. Also, the authors highlight the importance of translational research to develop accurate computational models of crop growth and development to help predict the effects of a changing climate on crop productivity [3].

## 4. Heading to the Sun: Vascular Growth and Developmental Changes in Shoot Architecture

Vascular development underlies every organogenesis and morphogenesis process to ensure resource delivery and mechanical support to any tissue and organ. Hellmann et al. [4] provide a comprehensive overview of the research on *Arabidopsis thaliana* vascular development and then focus on how this knowledge has been applied and expanded in research on the wood of trees and storage organs of crop plants. Basic principles of vascular development in roots, hypocotyl, leaves, and stems are reviewed, and gene regulatory networks involved are dissected and compared amongst models, woody species and Brassica crops, providing important hints on how to modulate cambial activity to improve productivity [4].

Translational biology from *Arabidopsis* to Brassica species is also the subject of the review from Leijten et al. [6] where the genetic networks involved in flowering time regulation in *Arabidopsis* are compared with related crop species in the Brassicaceae and with more distant vegetable crops within the Asteraceae family. Flowering time diversity has adaptive value in natural populations and plays a major role in agricultural production. In particular, it represents a crucial breeding trait for yield and nutritive quality of vegetable crops. This review is a collaboration among two public Institutions (the Italian CNR and the University of Amsterdam) involved in basic research, with the Research and Development group of Enza Zaden, an international vegetable-breeding company which develops new vegetable varieties that are grown and consumed all over the world. As a result, fundamental and applied science views on flowering time regulation intertwine, providing a comprehensive overview of basic genetic principles, available alleles and quantitative trait loci (QTL) and new perspectives for breeding strategies [6]. An overview of the molecular mechanisms of the shoot transition from juvenility to adult phases and flowering in fruit tree species can be found in the last section of De Paolis et al. [1].

A useful allele that can be used for wheat breeding programs to develop semi-dwarf cultivars is described in an article by Grant et al. [7]. The introduction of semi-dwarf varieties, that are more responsive to changing agriculture practices, was important during the green revolution in the mid-twentieth century to increase cereal production. Grant et al. report the inheritance and genetic mapping of the *Reduced Height 18 (Rht18*) gene in wheat and the selection of a semi-dwarf line with superior agronomic characteristics that could be utilized in breeding programs [7].

The genetic pathways that plants activate to sense and react to the presence of neighboring plants in the shade avoidance response is reviewed in Sessa et al. [5]. The authors critically summarize the current knowledge on the multiple pathways and regulators involved in this adaptive process, that can result in phenotypes with a high relative fitness in individual plants growing within dense vegetation. Recent advances in the molecular description of the shade avoidance response in crops, such as maize and tomato, and their similarities and differences with *Arabidopsis*, are discussed together with strategies to attenuate shade avoidance at defined developmental stages and/or in specific organs in high-density crop plantings [5].

## 5. Plant Cell Culture: Powerful Tools for Biotechnology

Most crops are recalcitrant to genetic transformation and/or regeneration; this represents a bottleneck in applying genome editing (GE) technologies to enhance crop productivity. In their review, Gordon-Kamm et al. [9] from the Agriculture Division of DowDuPonts (Corteva Agriscience company, Dupont Pioneer) provide an overview on how ectopic overexpression of genes involved in morphogenesis could and have been used to improve transformation efficiencies of recalcitrant crops. These genes are mainly regulators of embryo and meristem formation, or involved in hormonal pathways, and are discussed by the authors based on their practical or potential benefit when used for transformation. Due to their important function in plant growth and development, constitutive or strong expression of these genes often cause undesired pleiotropic effects. Gordon-Kamm et al. share with the readers the many possible strategies to limit/overcome pleiotropic deleterious problems, providing examples from the literature and from their own in-house experience in cereal crops [9]. These strategies might be applied to most recalcitrant crop species, including crop legume species that are mainly recalcitrant to in vitro culture and for which high throughput genetic transformation systems are yet to be developed. This is highlighted in the section dedicated to the genetic transformation of legumes in De Paolis et al. [1], where the power of in vitro plant cell and tissue cultures for applied biotechnology is also reviewed in the first section.

## 6. Conclusions

The knowledge acquired so far on the genetic basis of plant development, and its great potential in crop science and breeding to improve the yield and quality of agricultural products, are summarized in this Special Issue. Several target genes and pathways for root and shoot design are available for application in precision breeding to improve performance and productivity of crops, and more will come in the near future with the increase of translational research in plants. The readers will find several hints, molecular tools, and strategies to translate plant development basic research into crop productivity traits.
